# The impact of a diabetes diagnosis on health and well‐being: Findings from the English Longitudinal Study of Ageing

**DOI:** 10.1111/1753-0407.13518

**Published:** 2023-12-19

**Authors:** Camilla Böhme Kristensen, Joseph Chilcot, Sarah E. Jackson, Andrew Steptoe, Ruth A. Hackett

**Affiliations:** ^1^ Health Psychology Section, Institute of Psychiatry, Psychology and Neuroscience King's College London London UK; ^2^ Department of Behavioural Science and Health, Institute of Epidemiology and Health Care University College London London UK

**Keywords:** diabetes, diagnosis, health, quality of life, well‐being

## Abstract

**Background:**

Poorer health and well‐being are associated with diabetes risk. However, little is known about the trajectory of health and well‐being from before to after diabetes diagnosis. We compared depressive symptoms, quality of life, self‐rated health, and loneliness at three time points (prediagnosis, diagnosis, 2–4 years post diagnosis) in individuals who developed diabetes and a comparison group.

**Methods:**

Health and well‐being measures were self‐reported by 3474 participants from the English Longitudinal Study of Ageing. Repeated measures analysis of variance and generalized estimating equations were used to investigate differences by group, time, and group‐by‐time interactions.

**Results:**

A total of 473 (13.6%) participants developed diabetes. The diabetes group reported greater depressive symptoms (*W* ^*2*^(1) = 20.67, *p* < .001) and lower quality of life (*F* = 1, 2535 = 10.30, *p* = .001) and were more likely to rate their health as fair/poor (*W* ^*2*^(1) = 67.11, *p <* .001) across time points, adjusting for age, sex, and wealth. They also reported greater loneliness (*F* = 1, 2693 = 9.70, *p* = .002) in unadjusted analyses. However, this was attenuated to the null in adjusted analyses. The group‐by‐time interaction was significant for quality of life (*F =* 1.97, 5003.58 = 5.60, *p =* .004) and self‐rated health (*W* ^*2*^(2) = 11.69, *p =* .003), with a greater decline in these measures over time in the diabetes group in adjusted analyses.

**Conclusion:**

People who received a diabetes diagnosis had greater depressive symptoms, lower quality of life, and poorer self‐rated health than those who did not develop diabetes. Quality of life and self‐rated health deteriorated more rapidly following a diagnosis. Screening for these factors around the time of diagnosis could allow for interventions to improve the health and well‐being of those with diabetes.

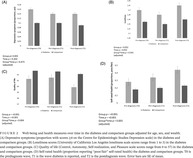

## Graphical abstract

- People who develop diabetes report greater depressive symptoms and lower quality of life and rate their health as poorer than those who do not develop diabetes.
- Around the time of diagnosis, people with diabetes experience a greater decline in quality of life and self‐rated health than adults who do not develop the condition.

## 1 INTRODUCTION

Type 2 diabetes (T2D) is a chronic metabolic condition that represents a major public health challenge globally.^1^ As the prevalence of T2D has increased,^1^ so has recognition in diabetes care guidelines^2^, ^3^ and reviews^4^ that self‐reported indicators of health and well‐being play a role in the condition.

The most commonly researched aspect of well‐being in diabetes is depression,^4^ with several meta‐analytic studies demonstrating that on average, people with T2D report more depressive symptoms than those without T2D.^5^, ^6^ Other health and well‐being factors are less well studied and understood,^4^ but there is some evidence to suggest that T2D may be associated with higher levels of loneliness and poorer quality of life (QoL). For example, in a study of over 4000 participants from the English Longitudinal Study of Ageing (ELSA) cohort, loneliness was more prevalent in those with than without T2D.^7^ Other work in an Australian study of 26 344 participants suggests that the odds of reporting poorer QoL is 49% higher among people with than without T2D.^8^ As well as poorer ratings of health and well‐being, there is evidence that people with T2D may also rate their overall health as poor. For example, in a recent study of 97 691 European participants, T2D was associated with poor self‐rated health.^10^ Specifically, 62.7% of those with T2D rated their health as poor compared with 33% of adults who were diabetes free.

Poor health and well‐being are also suggested to increase the risk of incident T2D in initially healthy populations. For example, in a pooled analysis of 497 223 T2D‐free participants, those who reported depression had a 25% increased risk of T2D over a 5‐year follow‐up.^9^ Recent evidence suggests that loneliness may also be a risk factor for T2D onset. For instance, in the ELSA cohort, loneliness was associated with incident T2D, independent of depression, sociodemographic factors, and health behaviors.^7^ This was confirmed in a subsequent Danish study, where loneliness was associated with incident T2D 5 years later.^10^

Poorer QoL may likewise be involved in T2D onset, as a prospective study of 5367 German participants showed that people who developed T2D had poorer QoL at baseline than those who did not develop T2D over 8.7 years follow‐up.^11^ Self‐rated health also been implicated in T2D risk. An early investigation of 7348 participants from Australia and New Zealand^12^ found that after adjusting for diabetes risk factors, including depression, self‐rated health was a predictor of incident T2D. Similar associations between self‐rated health and T2D risk have been observed in a sample of 250 805 Korean participants.^13^

Although most studies to date have focused on ratings of health and well‐being in relation to T2D risk, increasing work suggests these factors may also influence prognosis in people with existing T2D. For instance, pooled prospective evidence indicates that depression is linked with the microvascular (eg, retinopathy, nephropathy, neuropathy) and macrovascular complications of diabetes.^14^, ^15^ Furthermore, depression in people living with T2D is associated with increased risk of hospitalization^15^ and mortality.^16^ Limited work has looked at loneliness in relation to diabetes complications. However, one recent longitudinal study of 2934 participants with diabetes found that higher levels of loneliness were associated with higher levels of functional limitations in diabetes.^17^ Poor QoL has also been implicated in T2D with a review suggesting that worse QoL was associated with poorer control of cardiovascular risk factors.^18^ Low self‐rated health has also been implicated in diabetes prognosis. In a prospective study with 7348 participants, low self‐rated health was associated with an increased risk of heart failure, lower extremity ulcers, amputation, and renal dialysis.^12^

Taken together, the evidence to date suggests that people with T2D rate their health and well‐being as poorer when compared to people without the condition.^5^, ^6^, ^19^, ^20^ The evidence also suggests that well‐being and self‐rated health may increase the risk of T2D in initially healthy populations^6^, ^7^, ^10^, ^11^, ^21^ and may influence prognosis (such as the onset of diabetes complications^22^) in those with overt diabetes.^23^

However, little is known about the trajectory of well‐being and self‐rated health over time from before to after T2D diagnosis as prospective studies have either (a) tracked health and well‐being in initially healthy individuals to the time of T2D diagnosis or (b) have tracked changes in these factors in the postdiagnosis period alone.

There is evidence from other health conditions such as cancer^24^, ^25^ that additional insights may be garnered from monitoring health and well‐being from the pre‐ to the postdiagnosis period in those who do and do not develop the condition of interest. However, to date, no studies have examined well‐being and health in the years leading up to T2D diagnosis, at the time of diagnosis, and in the years following diagnosis.

In light of this evidence gap, the current study set out to investigate changes in depressive symptoms, loneliness, QoL, and self‐rated health across three time points: 0–2 years before diabetes diagnosis, 0–2 years during diagnosis and 2–4 years post diagnosis in adults receiving a new diagnosis of T2D and a healthy comparison group.

## 2 METHODOLOGY

### 2.1 Sample

Data were from the ELSA cohort, a representative sample of men and women aged >50 years residing in England.^26^ The study commenced in 2002–2003 (wave 1) with 12 099 participants. The study has since continued biannually. The data collection consists of self‐reported questionnaires and computer‐assisted interviews, with objective biomarker data collected at alternate study waves. Ethical approval was obtained from the National Research Ethics Committee.

**FIGURE 1:**
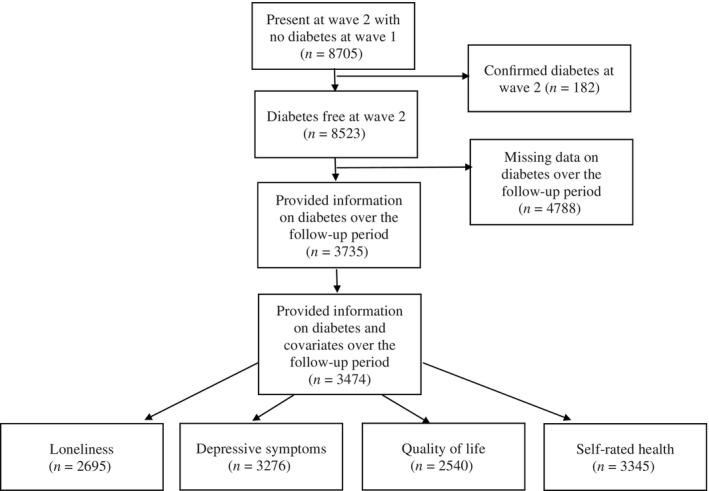
Flow chart of participants included and excluded from the study. To obtain the analytic sample only participants who were diabetes free at wave 2 and participants who had psychosocial data at three consecutive waves for at least one psychosocial factor of interest were included.

The present study uses data beginning from wave 2 (2004–2005) as loneliness was first measured in wave 2. We were interested in three time points in this study; prediabetes diagnosis (which was defined as T0), peridiabetes diagnosis (the wave diabetes was first reported; defined as T1), and postdiabetes diagnosis (defined as T2). The prediagnosis wave was defined as the wave preceding the diabetes diagnosis. Participants who first reported a new diagnosis of T2D between waves 3 (2006–2007) and 7 (2014–2015) were classified as diabetes cases. Participants with an existing diagnosis of T2D at wave 2 (T0) were excluded. Participants who received a new diagnosis of T2D in wave 8 (2016–2017) were excluded as there was no postdiagnosis wave for these participants. The comparison group data was taken at waves 4 (2008–2009) to 6 (2012–2013) and consisted of participants who did not report a T2D diagnosis in any wave and who had data on at least one health and well‐being outcome for three consecutive waves. The participant selection process is detailed in Figure 1 and resulted in a final sample size of 3474 participants.

### 2.2 Diabetes diagnosis

At each wave, participants were asked whether a physician had given them a diagnosis of diabetes or high blood sugar since the previous wave.

### 2.3 Health and well‐being measures

Depressive symptoms were measured using the eight‐item Centre for Epidemiologic Studies Depression Scale. Participants were asked whether they had experienced depressive symptoms such as “I felt depressed” and “My sleep was restless” in the past month with binary response options of yes/no. Scores can range from 0 to 8 with a higher score indicating greater depressive symptoms.^27^ As the data were skewed, we created a binary variable using an established cutoff (≥4) to indicate depressive symptomatology.^28^

Loneliness was measured using the three‐item Revised University of California Los Angeles Loneliness Scale (UCLA). The three questions “How often do you feel you lack companionship?,” “How often do you feel left out?,” and “How often do you feel isolated from others?” are included in this scale. Responses range from 1 = “hardly ever” to 3 = “often.” Scores were averaged and ranged from 1 to 3 with a higher score indicating greater levels of loneliness.^29^

QoL was measured with the 19‐item Control, Autonomy, Self‐realisation, and Pleasure (CASP) scale. Examples of questions included in this scale are “I feel free to plan for the future” and “I feel that my life has meaning.” A four‐point Likert response ranging from 0 = “never” to 3 = “often” is included in this scale. Scores can range from 0 to 57 with a higher score indicating greater levels of QoL.^30^

Self‐rated health was assessed with the single item question “In general, would you say your health is 1 = excellent, 2 = very good, 3 = good, 4 = fair, and 5 = poor.”^31^ Self‐rated health was used as a binary variable with “poor/fair” versus “excellent/very good/good” self‐rated health. This derived variable has been used in previous ELSA studies.^32^

### 2.4 Other measures

Age (years), sex (male/female), and wealth at T0 were included as covariates in the analyses as they are influential factors in diabetes.^33^ Ethnicity was categorized as “White,” and “ethnic minority,” and marital status was defined as “single,” “married,” “separated/divorced,” and “widowed.” Wealth was assessed with nonpension wealth, which is the most relevant indicator of socioeconomic status in this cohort. Nonpension wealth was presented in quintiles (1 = low to 5 = high) derived for the entire wave.^34^ Glycated hemoglobin (HbA1c) was assessed from blood drawn from participants' forearms during the ELSA nurse visit at wave 4 (2008–2009). Those who reported having a clotting or bleeding disorder or that they were taking anticoagulant medication did not provide blood samples. Some of the participants included in our sample were missing data on HbA1c (see Table 1). Height and weight were objectively measured at the wave 4 (2008–2009) nurse visit. This information was used to calculate body mass index (BMI; kg/m^2^). Participants self‐reported whether they had ever received a diagnosis of high blood pressure or hypertension (yes/no) at every wave. Self‐reported information on diagnoses of myocardial infarction and angina were also recorded each wave. This information was combined to create a measure of coronary heart disease (CHD; yes/no). In addition, self‐reported information on stroke diagnosis (yes/no) was collected at all time points.

**TABLE 1:** Participant characteristics.

Characteristic	*N*	Diabetes group (*n =* 473)	*N*	Comparison group (*n =* 3001)	*p* value
Age (years)	473	68 (13)	3001	65 (11)	<.001
HbA1c (%)	271	6.63 (0.99)	2171	5.72 (0.34)	<.001
Sex (% men)	473	245 (51.8%)	3001	1262 (42.1%)	<.001
Ethnicity (% White)	472	454 (96.2%)	3001	2954 (98.4%)	<.001
Wealth (%)	473		3001		<.001
1 (lowest)		131 (27.7%)		358 (11.9%)	<.001
2		104 (22.0%)		496 (16.5%)	
3		95 (20.1%)		657 (21.9%)	
4		64 (13.5%)		686 (22.9%)	
5 (highest)		79 (16.7%)		804 (26.8%)	
Married (% yes)	473	182 (38.5%)	3001	919 (30.6%)	<.001
Body mass index (kg/m^2^)	351	30.90 (5.72)	2557	27.84 (4.78)	<.001
Coronary heart disease (% yes)	470	84 (17.9%)	2944	227 (7.7%)	<.001
Hypertension (% yes)	470	299 (63.6%)	2944	1271 (43.2%)	<.001
Stroke (% yes)	473	34 (7.2%)	3001	97 (3.2%)	<.001

*Note*: Age and glycated hemoglobin (HbA1c) are presented as median (interquartile range). Body mass index is presented as mean (SD). Other variables are presented as number (percentage).

### 2.5 Statistical analyses

The descriptive characteristics of the sample are reported as medians (interquartile range) and means (SDs), for continuous variables and numbers (percentages) for categorical variables. At baseline (T0, the wave preceding T2D diagnosis), Pearson's chi‐square test was used to compare the following categorical variables: sex, ethnicity, nonpension wealth, marital status, CHD, hypertension and stroke between those who reported T2D over the study period and the comparison group. Age and HbA1c were skewed, hence the Mann–Whitney *U* test was used to compare age and HbA1c between those who reported T2D and the comparison group. Independent t‐test was used to compare BMI between the groups. Repeated analysis of variance was used for analyses of the continuous variables (loneliness and QoL) and generalized estimating equations were used for analyses of the categorical variables (depressive symptoms and self‐rated health). Analyses tested the main effect of time (within‐subject analysis), that is, changes in health and well‐being over time, independent of group (diabetes versus comparison) and the main effect of group, that is, the overall difference between the diabetes group and the comparison group in health and well‐being, independent of time and the group by time interaction, that is, whether changes in health and well‐being over time varied depending on group. Unadjusted and adjusted analyses are presented. Age, sex, and nonpension wealth were included as covariates in adjusted analyses. We conducted several sensitivity analyses to see whether BMI or the physical comorbidities of CHD, hypertension or stroke prior to diabetes diagnosis influenced the patterning of our results. Each condition was looked in separately and was added as an additional covariate to the models. BMI was taken at wave 4 (2008–2009) for both the diabetes and comparison groups. Information on CHD, hypertension, and stroke was included at the wave prior to diagnosis for diabetes group (T0) and at wave 4 (2008–2009) for the comparison group. The analyses were conducted in IBM SPSS version 25.

## 3 RESULTS

### 3.1 Sample characteristics

Table 1 details the sample characteristics of 3474 individuals who took part in the study. Of these 473 (13.6%, 95% confidence interval [CI] = 12.5%–14.8%) developed diabetes. The participants who developed diabetes were older on average (median = 68 years; interquartile range [IQR] = 13 vs 65 years [IQR = 11], *p* < .001), were more likely to be male, (*x* ^*2*^(1) = 15.80, *p* < .001), had lower nonpension wealth (*x* ^*2*^(4) = 112.66, *p* < .001), and were more likely to be married (*x* ^*2*^(1) = 11.65, *p* < .001) and from an ethnic minority group (*X* ^*2*^(1) = 11.22, *p <* .001), compared to those who did not develop diabetes. HbA1c levels were higher on average in the diabetes group (median = 6.63% [IQR = 0.99] vs 5.72% [IQR = 0.34], *p* < .001).

### 3.2 Well‐being and health in the diabetes and comparison groups

**FIGURE 2:**
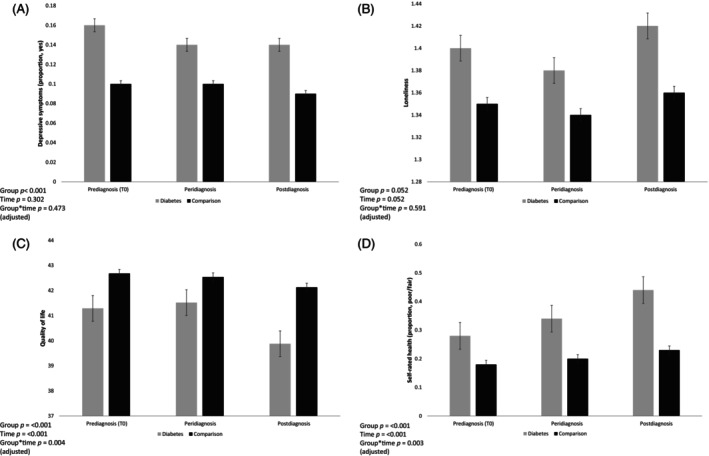
Well‐being and health measures over time in the diabetes and comparison groups adjusted for age, sex, and wealth. (A) Depressive symptoms (proportion with scores ≥4 on the Centre for Epidemiologic Studies Depression scale) in the diabetes and comparison groups. (B) Loneliness scores (University of California Los Angeles loneliness scale scores range from 1 to 3) in the diabetes and comparison groups. (C) Quality of life (Control, Autonomy, Self‐realization, and Pleasure scale scores range from 0 to 57) in the diabetes and comparison groups. (D) Self‐rated health (proportion reporting “poor/fair” self‐rated health) the diabetes and comparison groups. T0 is the prediagnosis wave, T1 is the wave diabetes is reported, and T2 is the postdiagnosis wave. Error bars are SE of mean.

Table 2 details ratings of well‐being and health over time for the diabetes and comparison groups. Depressive symptoms did not change significantly over the study period controlling for age, sex and wealth (*W* ^*2*^(2) = 2.39, *p =* .302). There was no significant effect of age on depressive symptoms over time (*W* ^*2*^(1) = 2.81, *p =* .093). However, the groups differed in depressive symptoms on average across time points (*W* ^*2*^(1) = 20.67, *p <* .001), with greater depressive symptoms observed in the diabetes group after adjusting for age, sex, and wealth. No significant group‐by‐time interaction was detected in adjusted analyses, (*W* ^*2*^(2) = 1.50, *p =* .473; Figure 2A), indicating that the change in depressive symptoms over the study period did not vary by group.

**TABLE 2:** Health and well‐being factors over the study period for the diabetes and comparison group.

Health and well‐being factor		Diabetes group				Comparison group					Time	Group	Group X time
	*n*		T0	T1	T2	*n*	T0	T1	T2		*p* value	*p* value	*p* value
Depressive symptoms (yes)	376	Proportion (SE)	0.20 (0.02)	0.17 (0.02)	0.17 (0.02)	2900	0.11 (0.01)	0.11 (0.01)	0.10 (0.01)	Unadjusted	.308	.001	.471
		Proportion (SE)	0.16 (0.02)	0.14 (0.02)	0.14 (0.02)		0.10 (0.01)	0.10 (0.01)	0.09 (0.01)	Adjusted	.302	.001	.473
Loneliness	273	Mean (SE)	1.43 (0.03)	1.41 (0.03)	1.45 (0.03)	2422	1.34 (0.01)	1.34 (0.01)	1.35 (0.01)	Unadjusted	.116	.002	.741
		Mean (SE)	1.40 (0.03)	1.38 (0.03)	1.42 (0.03)		1.35 (0.01)	1.34 (0.01)	1.36 (0.01)	Adjusted	.052	.052	.591
Quality of life	247	Mean (SE)	40.35 (0.52)	40.60 (0.52)	38.94 (0.53)	2293	42.78 (0.17)	42.64 (0.17)	42.23 (0.17)	Unadjusted	.001	.001	.003
		Mean (SE)	41.29 (0.50)	41.52 (0.50)	39.88 (0.50)		42.68 (0.16)	42.54 (0.16)	42.13 (0.17)	Adjusted	.001	.001	.004
Self‐rated health (fair/poor)	383	Proportion (SE)	0.32 (0.02)	0.38 (0.03)	0.48 (0.03)	2962	0.18 (0.01)	0.19 (0.0)	0.22 (0.01)	Unadjusted	.001	.001	.004
		Proportion (SE)	0.28 (0.02)	0.34 (0.02)	0.44 (0.03)		0.18 (0.01)	0.20 (0.01)	0.23 (0.01)	Adjusted	.001	.001	.003

*Note*: Binary variables: depressive symptoms (scores <4 vs >4) and self‐rated health (excellent/very good/good vs fair/poor) presented as proportions (SE). Continuous variables: loneliness (range 1–3) and quality of life (range from 0 to 57) presented as mean (SE). Adjusted analyses include age, sex, and wealth.

Loneliness levels did not significantly change over the study period (*F =* 1.96, 5279.51 = 2.97, *p =* .052) adjusting for age, sex, and wealth. No significant effect of age on loneliness over time was detected (*F =* 1.97, 486.98 = 1.72, *p =* .179). In unadjusted analyses, there was a main effect of loneliness, with higher levels observed in the diabetes group (*F =* 1, 2693 = 9.70, *p =* .002). However, in adjusted analyses loneliness levels (*F =* 1, 2690 = 3.79, *p =* .052) did not significantly differ between the diabetes and comparison groups (although mean scores tended to be higher on average in the diabetes than in the comparison group). Further, no significant group‐by‐time interaction was detected for loneliness (*F =* 1.96, 5279.51 = 0.52, *p =* .591; Figure 2B) in adjusted analyses.

A significant main effect of time was detected for QoL (*F =* 1.97, 5003.58 = 15.65, *p <* .001), suggesting that QoL decreased on average over the study period. The main effect of group was also significant (*F* = 1, 2535 = 10.30, *p <* .001), suggesting that average QoL in the diabetes group were lower than in the comparison group, where lower scores represent worse QoL. The group‐by‐time interaction was also significant (*F =* 1.97, 5003.58 = 5.60, *p =* .004), suggesting that mean scores over time differed between the two groups; with the diabetes group having lower QoL throughout the study period with a greater decline over time seen in the diabetes group only (Figure 2C). These associations were independent of age, sex, and wealth. In addition, a significant effect of age on QoL over time was detected (*F =* 1.97, 88 405 = 23.59, *p <* .001). However, all QoL findings were robust to adjustment for age.

A significant main effect of time (*W* ^*2*^(2) = 65.06, *p <* .001) was observed for self‐rated health, with the proportion of participants classifying their self‐rated health as fair/poor increasing over time. The groups significantly differed in self‐rated health on average over the study period, (*W* ^*2*^(1) = 67.11, *p* < .001) and a significant interaction between time and group was established, (*W* ^*2*^(2) = 11.69, *p* = .003), suggesting that the change in self‐rated health scores over time differed between the two groups. The proportion of participants rating their self‐rated health as fair/poor over the study period was higher in the diabetes group, suggesting that self‐rated health became worse in people with diabetes over time (Figure 2D). These findings were robust to adjustment for age, sex, and wealth.

We also observed a significant effect of age on self‐rated health over time (*W* ^*2*^(1) = 43.42, *p <* .001). However, all SRH findings were robust to adjustment for age.

### 3.3 Sensitivity analyses

We ran a series of sensitivity analyses to see whether our findings were similar when adjusting for BMI, as well as the comorbid physical conditions of hypertension, CHD, or stroke. As can be seen in Table S1, the findings for depressive symptoms and self‐rated health were unaltered when taking the three comorbidities or BMI into account. The findings for loneliness and quality of life were also similar when adjusting for hypertension, CHD, and stroke.

However, the inclusion of BMI in the models moved the increase in loneliness over time, as well as the trend for greater loneliness in diabetes than the comparison group to statistical significance (*p* = .026 and *p* = .038, respectively). The inclusion of BMI also moved the group difference in QoL to nonsignificance (*p* = .071). However, the group‐by‐time interaction for QoL remained robust to adjustment for BMI.

## 4 DISCUSSION

The current study investigated the impact of a diabetes diagnosis on the health and well‐being of community dwelling sample of middle‐aged and older adults. We found that those who received a diagnosis of diabetes were more likely to report greater depressive symptoms, lower QoL, and poorer self‐rated health than those who did not report diabetes across assessments taken before, around the time of, and after their diagnosis. In unadjusted analyses, those with diabetes reported greater levels of loneliness though these findings were attenuated in adjusted models. QoL and self‐rated health deteriorated more rapidly over time in those who received a diabetes diagnosis, adjusting for age, sex, and wealth. No other significant group‐by‐time interactions were observed. The findings were similar when BMI and cardiovascular comorbidities were included in the models.

Our finding that those who received a diabetes diagnosis reported greater depressive symptoms is in line with previous research. Several meta‐analytic studies have shown elevated depressive symptoms in people with T2D, in comparison to healthy individuals.^5^, ^6^ In the present study, the reporting of depressive symptoms over the study period remained stable in both the T2D and comparison group. Depressive symptoms were higher in the T2D group at the wave before their diagnosis, indicating that these symptoms emerge before overt disease is recognized and diagnosed. Clarifying how long before a diagnosis such differences in depressive symptomatology emerge is an avenue for future work. We did not find a significant group‐by‐time interaction for depressive symptoms, which is in line with previous research on cancer using the ELSA cohort.^25^ Williams and colleagues examined health and well‐being from before, during, and after cancer diagnosis in people who developed cancer and a cancer‐free comparison group, where a nonsignificant effect of group‐by‐time was found.^25^ This suggests that receiving a new diagnosis of T2D or cancer or adjusting to life after a diagnosis does not appear to lead to a change in depressive symptoms in middle‐aged and older adults in this cohort.

People who received a new diagnosis of T2D reported higher levels of loneliness compared to healthy comparisons in unadjusted analyses, though the observed group difference only approached significance (*p* = .052) when taking age, sex, and wealth into account. This suggests that on average loneliness values tend to be higher in people with T2D than in the comparison group, though this difference is attenuated when demographic factors are considered. This near significant finding is in line with previous work in this cohort,^7^ where higher loneliness scores were also observed in people with diabetes compared to people without diabetes in unadjusted analyses. Loneliness in T2D is understudied,^7^ but some evidence on loneliness and diabetes is available. For example, a Danish cohort study found that loneliness was associated with T2D development 5 years later in people who were initially diabetes free.^10^ More research has focused on loneliness in cardiovascular disease (CVD), which is a leading cause of mortality in diabetes.^10^, ^35^ Evidence suggests that people with CVD are lonelier than those without CVD.^36^ Therefore, taking previous studies on T2D^10^ and CVD^36^ together the result from this study is somewhat unexpected, though loneliness values trended in the expected direction. It is possible we were underpowered to detect a significant effect in adjusted analyses, as these earlier studies using other cohort data^12^, ^41^ benefitted from larger sample sizes than in the current study.

We did not find any significant changes in loneliness over the study period among people who reported T2D and the comparison group. Likewise, no significant group‐by‐time interaction was established, suggesting there is not a significant change in loneliness levels around the time of diabetes diagnosis. No other research has looked at changes in loneliness in the period surrounding T2D diagnosis, which limits our ability to compare findings from this study with previous literature. It is possible that the lack of significant change in loneliness over time in the diabetes group could be due to increased social support by friends and family when an individual receives a serious diagnosis, which could mitigate expected changes in this measure. However, research is needed to test this assertion. In samples assessing healthy adults alone, whether levels of loneliness change at middle and older age is an area of contention, with some work suggesting no change^37^ and others observing distinct patterns for certain subgroups.^38^ In light of this mixed evidence in healthy groups and limited work in relation to T2D, further research is needed to look at possible changes in loneliness from the pre‐ to post‐T2D diagnosis period.

People who received a T2D diagnosis had significantly lower QoL on average than the comparison group. The greater prevalence of poor QoL in people with than without T2D has been observed in earlier^39^ and more recent work.^8^ An Australian cohort study of 26 344 participants suggested that ratings of poorer QoL were 49% higher amongst people with than without diabetes.^8^ The current study adds to this literature. Although ratings of QoL declined over time for both groups, the decline was steeper for those who received a T2D diagnosis (as evidenced by the significant group‐by‐time interaction). This suggests T2D diagnosis negatively affects QoL at a greater rate than the age‐associated declines in QoL seen in the comparison group. The decline in QoL around the time of T2D diagnosis may be unsurprising as these individuals had received a serious diagnosis.

QoL continued to decline in the postdiagnosis period for the T2D group, which could be attributed to the stress associated with living with a chronic condition.^40^ It is well established that diabetes management is very demanding.^2^, ^3^ The complexity of self‐care activities, the need for lifestyle change, fear of complications, along with the social impact of the condition may lead to frustration, stress, and discouragement, encapsulated by the term diabetes distress. This illness‐specific distress is a prominent issue in T2D.^41^, ^42^ Evidence suggests diabetes distress is associated with poor QoL.^43^ The decline in QoL observed following T2D diagnosis in this study may reflect such issues. Though further work is required to test this assertion as we did not have a measure of diabetes distress in this study.

The T2D group were significantly more likely to rate their health as poor or fair on average than those in the comparison group. It is plausible that poor/fair self‐rated health in the lead up to T2D diagnosis may reflect undiagnosed diabetes with symptoms relating to the condition presenting themselves. This is in line with previous evidence showing that 62.7% of people with T2D (in comparison to only 33% of people without diabetes) rated their health as poor in a sample of 97 691 participants.^44^ Self‐rated health changed significantly over the three time points, independent of group, indicating that more people rated their health as fair/poor over time. However, this change in self‐rated health was more considerable for those who received a T2D diagnosis. As indexed by a significant group‐by‐time interaction, the proportion of people reporting poor/fair self‐rated health increased more rapidly over the study in the diabetes group when compared with the healthy comparison group. The increase in poor/fair self‐rated health around the time of diagnosis could be expected after receiving news of a chronic illness. The proportion of those rating their health as poor/fair further increased in the postdiagnosis period for the T2D group.

The observed steep increase in poor/fair self‐rated health around the time of diagnosis is in accordance with previous research on self‐rated health in other long‐term conditions.^24^, ^25^ In these studies of cancer, those who received a cancer diagnosis were more likely to rate their health as fair/poor than a comparison group on average.^24^, ^25^ Similarly, in this earlier study a significant group‐by‐time interaction was established for self‐rated health, suggesting that the number of participants who rated their heath as fair/poor increased significantly more over time in those who received a cancer diagnosis than the comparison group.^27^

The significant increase in poor/fair self‐rated health following diagnosis T2D could be related to health behavior. Lifestyle modifications are critical in diabetes management and outcomes.^1^ However, evidence from this cohort suggests that receiving a T2D diagnosis is not a major cue to alter lifestyle behavior.^45^ Lack of engagement with recommended lifestyle changes following diagnosis could increase the risk of diabetes complications, which in turn would affect this population's self‐rated health. This might influence the increase in poor/fair self‐rated health in the postdiagnosis period. However, T2D is a progressive condition where pancreatic functioning continues to decline.^1^ Thus, the associated decline in self‐rated health the years after the T2D diagnosis may reflect natural disease progression. More research is needed to tease out these associations.

### 4.1 Strengths and limitations

To the best of our knowledge, this is the first study to examine the impact of a diabetes diagnosis on health and well‐being over time. It provides valuable insights into the trajectories of health and well‐being measures, from the time leading up to a diagnosis, to the years immediately post diagnosis. The study benefitted from the use of a large well‐defined cohort of middle‐aged and older adults. We were able to take advantage of repeated measurements of health and well‐being over seven waves of data collection in the ELSA cohort. The present study is not, however, without limitations. First, diabetes diagnosis was self‐reported, which may have resulted in missed cases of diabetes. However, evidence suggests a strong correlation between physician‐registered and self‐reported diabetes diagnoses.^46^ The precise date of diabetes diagnosis was unknown and could have happened any time in the 2 years between T0 and T1. Consequently, the measurements of health and well‐being factors in T1 were not at the same moment as diabetes diagnosis, but^55^ also fell in a 2‐year range. Furthermore, we had no information on the exact age of participants at diagnosis (because each ELSA wave covers a 2‐year period), the type of diabetes developed or whether participants had a family history of diabetes or whether they had comorbid chronic kidney disease. This meant we were unable to assess the influence of these variables on our results. Lastly, to investigate possible changes in health and well‐being factors over time, only participants with health and well‐being information for three consecutive waves were included in this study. This reduced our sample size and may have led to selection bias.

In conclusion, people who received a diagnosis of T2D were more likely to report greater depressive symtpoms, lower QoL, and fair/poor self‐rated health on average than those who did not develop diabetes. We also observed that these differences are present in the time before T2D diagnosis. This could offer the possibility that screening for these factors could be beneficial in the early detection of diabetes. The study also highlights the need for psychological support for people following a diabetes diagnosis, as self‐rated health and QoL appear to deteriorate around this time at a greater rate than observed in controls. Screening for these factors around the time of a T2D diagnosis could allow for targeted interventions to help minimize the impact of a diagnosis on the self‐reported health and well‐being of people with T2D.

## AUTHOR CONTRIBUTIONS

Camilla Böhme Kristensen: formal analysis, writing–original draft; Joseph Chilcot: writing–review and editing; Sarah E. Jackson: conceptualization, data curation, writing–review and editing; Andrew Steptoe: conceptualization, writing–review and editing; Ruth A. Hackett: supervision, Conceptualization, formal analysis, writing–original draft; writing–review and editing.

## FUNDING INFORMATION

None.

## DISCLOSURE

None.

## Supporting information


**Supplementary Table S1:** Health and well‐being factors over the study period for the diabetes and comparison group with additional adjustment.
