# Supplementary material for: The impact of a diabetes diagnosis on health and well‐being: Findings from the English Longitudinal Study of Ageing
Source: J Diabetes. 2023 Dec 19;16(7):e13518. doi: 10.1111/1753-0407.13518 (PMC11212344; doi:10.1111/1753-0407.13518)
Supplement: Supplementary file 1 — Supplementary Table S1: Health and well‐being factors over the study period for the diabetes and comparison group with additional adjustment. [file JDB-16-e13518-s001.docx]

| **Supplementary Table 1:** Health and wellbeing factors over the study period for the diabetes and comparison group with additional adjustment | | | | | | | |
| --- | --- | --- | --- | --- | --- | --- | --- |
| **Health & wellbeing factor** |  | **Time** | | **Group** | | **Group X time** | |
|  |  | *F* or *W^2^* | *p* | *F*  or *W^2^* | *p* | *F*  or *W^2^* | *p* |
| Depressive symptoms | Basic adjustment | 2.39 | 0.302 | 20.67 | 0.001 | 1.50 | 0.473 |
|  | + BMI | 1.12 | 0.571 | 15.31 | 0.001 | 1.45 | 0.484 |
|  | + CHD | 2.31 | 0.315 | 18.47 | 0.001 | 1.57 | 0.455 |
|  | + hypertension | 2.40 | 0.301 | 18.69 | 0.001 | 1.56 | 0.459 |
|  | + stroke | 2.38 | 0.304 | 20.17 | 0.001 | 1.49 | 0.474 |
| Loneliness | Basic adjustment | 2.97 | 0.052 | 3.79 | 0.052 | 0.52 | 0.591 |
|  | + BMI | 3.69 | 0.026 | 4.33 | 0.038 | 0.74 | 0.475 |
|  | + CHD | 2.68 | 0.070 | 3.22 | 0.073 | 0.52 | 0.592 |
|  | + hypertension | 2.65 | 0.072 | 3.23 | 0.073 | 0.49 | 0.612 |
|  | + stroke | 2.89 | 0.057 | 3.62 | 0.057 | 0.50 | 0.604 |
| Quality of life | Basic adjustment | 15.65 | 0.001 | 10.30 | 0.001 | 5.60 | 0.004 |
|  | + BMI | 14.46 | 0.001 | 3.26 | 0.071 | 5.40 | 0.005 |
|  | + CHD | 15.20 | 0.001 | 8.11 | 0.004 | 5.38 | 0.005 |
|  | + hypertension | 15.02 | 0.001 | 7.05 | 0.008 | 5.17 | 0.006 |
|  | + stroke | 15.67 | 0.001 | 9.44 | 0.002 | 5.64 | 0.004 |
| Self-rated health | Basic adjustment | 67.11 | 0.001 | 65.06 | 0.001 | 11.69 | 0.003 |
|  | + BMI | 57.71 | 0.001 | 23.46 | 0.001 | 11.31 | 0.004 |
|  | + CHD | 68.40 | 0.001 | 54.44 | 0.001 | 12.20 | 0.002 |
|  | + hypertension | 67.32 | 0.001 | 50.11 | 0.001 | 11.55 | 0.003 |
|  | + stroke | 67.49 | 0.001 | 61.41 | 0.001 | 11.90 | 0.003 |

*BMI- Body Mass Index; CHD= Coronary Heart Disease.

*F* presented for continuous variables (loneliness, quality of life). *W*^2^ presented for categorical variables (depressive symptoms, self-rated health)
